# Examining associations between mental health and Chronic Non-Communicable Diseases (C-NCDs) among older adults in Wakiso, Uganda

**DOI:** 10.1371/journal.pone.0293993

**Published:** 2024-06-17

**Authors:** Robert M. Bulamba, Fred Nalugoda, James Nkale, Godfrey Kigozi, A. Malachi Ochieng, Emmanuel Kyasanku, Stephen Watya, Vitalis Ofumbi Olwa, Alex Daama, Violet Nkwanzi, Deusdedit Kiwanuka, Stephen Mugamba, Grace Kigozi, Jennifer Wagman, Anna Mia Ekström, Gertrude Nakigozi, Amanda P. Miller

**Affiliations:** 1 Africa Medical and Behavioral Sciences Organization (AMBSO), Kampala, Uganda; 2 Karolinska Institute, Solna, Sweden; 3 East Tennessee State University, Johnson City, TN, United States of America; 4 University of California, Los Angeles, Los Angeles, CA, United States of America; 5 San Diego State University, San Diego, CA, United States of America; Florida State University, UNITED STATES

## Abstract

**Background:**

Globally, the prevalence of chronic non-communicable diseases **(**C-NCDs) and occurrence of multi-morbidity specifically, has been increasing and will continue to rise as life expectancy increases. The burden of mental health disorders has also been rising globally. In sub-Saharan Africa (SSA), literature on these health issues, which are interrelated, is scarce. This study assesses the prevalence of C-NCDs, and depressive and anxiety symptomology and examines the relationship between these issues among a sample of older adults in Uganda.

**Methods:**

Between 2021–2022, 604 consenting adults aged 35 years and older were surveyed on a broad range of health issues for the ongoing AMBSO Population Health Surveillance (APHS) cohort study in Wakiso district. Descriptive analyses were performed to characterize the burden of C-NCDs (e.g. diabetes, hypertension), depression (PHQ-9 using a cutoff of <5 scores for minimal/no and 5+ for mild to severe symptomology) and anxiety (GAD-7 using a cutoff of 5+ scores for mild to severe symptomology). Bivariate analysis and multivariable logistic regression models were built using STATA software version 16.0 to examine associations between mental health disorders and having at least one C-NCD. Our exposures of interest were depressive and anxiety symptoms and our outcomes of interest was presence of C-NCDs.

**Results:**

Majority of participants were females (63.6%), median age was 46 (IQR: 39–54). Any C-NCDs prevalence was 18.7%, while 18.9% and 11.4%, had screening scores indicative of depressive and anxiety symptomology, respectively. Three percent (3.2%) had PHQ-9 scores indicative of moderate to severe depressive symptomology. In models adjusted for sociodemographic characteristics, there was 12% increased odds of suffering from C-NCDs for every unit increase in PHQ-9 score (AOR = 1.12, 95% CI: 1.10–1.20). Participants with any anxiety symptoms had 2.1 greater odds of suffering from C-NCD compared to those who did not have anxiety symptoms (AOR = 2.10, 95% CI: 1.21–3.70).

**Conclusion:**

C-NCDs were prevalent in older adults, particularly among those experiencing mental health symptoms. Screening for C-NCDs and mental health disorders should be integrated into routine health care for older adults in the country. Early screening and identification of these health issues through primary health care could significantly reduce the public health burden attributable to mental health disorders and the incidence of multi-morbidity in Uganda.

## Introduction

Reports from the World Health Organization (WHO) suggest that the global prevalence of chronic non-communicable diseases (C-NCDs) has risen over the past decade, particularly in low and middle-income countries (LMICs) [1]. As the global population continues to age, the total number of people affected by C-NCDs is expected to continue to rise, increasing by an estimated 27% in the African region over the next decade [2]. Globally, approximately 41 million people (~74% of all deaths) succumb to C-NCDs annually [3]. Seventeen million of these deaths occur before the age of 70 years; the overwhelming majority (86%) of these premature deaths occur in LMICs [22]. Collectively, C-NCDs are responsible for 7.4% of the global disability adjusted life years (DALYs) and 22.9% of years lived with disabilities (YLDs), making them the fifth leading cause of DALYs and YLDs, respectively [4]. The most prevalent C-NCDs in LMICs include cardiovascular diseases, cancers, chronic respiratory diseases, hypertension, stroke, type 2 diabetes mellitus, and chronic kidney disease [5]. While these trends mirror those found in high income countries (HICs), contextual drivers of C-NCDs in LMICs such as widespread poverty and limited access to C-NCD prevention strategies (healthy dietary choices, physical activity) and equitable healthcare suggest that intervention strategies that are contextually responsive are needed.

Although initially associated with HICs, the presence of C-NCDs has become the dominant health burden in many LMICs, including those located in sub-Saharan Africa (SSA). Economic growth in SSA and intensive public health efforts targeting routine immunization and HIV prevention and treatment have fueled an epidemiologic transition from a primarily communicable to non-communicable burden of disease. Today, many individuals residing in LMICs have improved life expectancy, diets high in processed foods and more sedentary lifestyles than their predecessors. Urbanization has also led to increased exposure to air pollution. Collectively these changes have increased risk of “lifestyle diseases” such as heart disease, stroke, diabetes, obesity, metabolic syndrome, chronic obstructive pulmonary disease, and several types of cancer (e.g. lung, prostate [6]) [7]. Further, these conditions which have shared risk factors, tend to cluster; consequently, multimorbidity, which is associated with reduced quality of life among persons with C-NCDs, is also on the rise in LMICs [8]. There is also heterogeneity in the epidemiology of C-NCD risk within LMICs. A complex constellation of social, environmental, economic and behavioral risk factors results in differential risk; unsurprisingly, those experiencing poverty are most vulnerable and disproportionally burdened. Having a C-NCD has also been identified as a risk factor for falling into poverty in this setting (due to medical costs and loss of ability to work) [9]. This growing public health burden attributable to C-NCDs in LMICs seriously threatens national efforts to promote economic growth and public health efforts. Given the double burden of disease that African nations now face (ongoing acute infectious disease epidemics coupled with the emergence of C-NCDs), the anticipated further increase in the prevalence of C-NCDs [10] holds important implications for future health care policies and resource allocation in these settings. This upward trend is projected to continue as epidemiologic profiles and age structures of LMICs further shift. For example, the scale-up of life extending antiretroviral therapy for persons living with HIV- a tremendous public health accomplishment- has extended life expectancies, producing an aging population which will exacerbate the burden of C-NCDs [11]. Furthermore, evidence from a systematic review of the literature on the impact of the COVID-19 pandemic illustrated that concomitant chronic diseases present a major public health threat (i.e., excess morbidity and mortality) in the context of infectious disease pandemics as individuals experiencing them are more vulnerable to severe infection and poor prognosis [12].

The global burden of mental health (MH) disorders is also growing and the association between adverse mental and physical health outcomes is well documented in HICs [13]. Depression, the most prevalent MH disorder, affects nearly 300 million people globally, and its prevalence has increased more than 18% between 2005–2015 [4]. The majority of those living with mental health disorders (80%) reside in LMICs [14]. Despite the public health burden attributable to MH disorders in LMICs, MH services are scarce [15]. The relationship between mental and physical health is reciprocal and there is significant overlap in the diagnosis of MH disorders and C-NCDs, globally and in LMICs, specifically [16]. A robust body of literature suggests that MH disorders (e.g. depression, anxiety) are risk factors C-NCDs and can exacerbate C-NCD prognoses [17, 18]. Similarly, having a C-NCD is associated with subsequent mental health disorders [19]. There is also evidence that in the context of poverty, MH disorders and C-NCDs constitute a syndemic [19].

While global burden of disease estimates for the prevalence of C-NCDs, depression and anxiety disorders have been calculated, a number of these estimates for LMICs are based on single-site studies [13], which affects their external validity and limits the application of the research findings beyond the particular countries or regions represented in the samples. Further, despite the tremendous public health burden attributable to MH disorders and C-NCDs (collectively, non-communicable diseases) in SSA, limited research on the relationship between these health issues in this setting exists. The growing burden of C-NCDs in SSA and Uganda in particular, is both a health equity and a sustainable development issue as C-NCDs disproportionately burden persons of lower socio-economic status (SES) and are both a cause and a consequence of poverty [20]. Poverty is closely linked with C-NCDs and depression and anxiety disorders are predicted to impede poverty reduction initiatives in LMICs, particularly by increasing household costs associated with healthcare and treatment for these diseases [22]. Therefore non-communicable diseases threaten progress towards the 2030 agenda for sustainable development (SDGs (1) “End poverty in all its forms everywhere” and (3) “Ensure healthy lives and promote well-being for all at all ages”) [21] by leading to premature deaths and morbidity, and contributing to extreme poverty [22]. MH disorders are a serious and often debilitating form of NCD with far-reaching consequences on quality of life and socioeconomic attainment, that can have an intergenerational impact on individuals and their families [23]. The co-existence of MH disorders and C-NCDs leads to greater disability and places a tremendous burden on the healthcare systems of LMICs in addition to reducing productivity and impacting morbidity and mortality [24]. In order to provide estimates that can inform public health programming and integration of services to address non-communicable diseases in Uganda into existing care, the present study assesses the burden of C-NCDs and their relationship with prevalent MH disorders in a large community-based sample of older adults in Wakiso District.

## Materials and methods

### Study design, Study population and setting

The present study was conducted within the Africa Medical and Behavioral Sciences Organization (AMBSO) Population Health Surveillance (APHS), an open community-based longitudinal cohort study whose methods have been described previously in the cohort protocol paper [25]. In brief, the cohort started in 2018 and conducts annual data collection as well as mid-year census and surveys across six rural, urban, and semi-urban communities located in two Ugandan districts (Hoima and Wakiso). Residents aged 13 years and above who provide informed consent (or assent for minors) are eligible to participate in the survey. The main goal of the cohort is to generate data longitudinal data on health behavior and outcome trends through assessment and monitoring of HIV and other sexually transmitted infections (STIs), tuberculosis, reproductive and general health, sexual behaviors, gender-based violence, other infectious diseases, C-NCDs, genetic diseases, neglected tropical diseases, MH disorders, emerging diseases, family and population structures, environmental, socio-economic, and other health and health systems related information in Uganda.

The communities included in this study were selected through a community mapping exercise which compared population structure of multiple districts with different distances from urban centers and varying degrees of cultivation for their representativeness of three different community types (urban, semi-urban and rural) to improve the generalizability of the cohort’s to Uganda’s broader population. Criteria for identifying eligible communities for each community type included density of homes, number of shops, presence of bars, other social spaces and fueling stations, primary materials used to construct dwellings and the presence of paved roads. These attributes were selected because they were thought to be characteristic of communities in Uganda more broadly, increasing the representativeness of the cohort.

Data collection included a household census and enumeration of the community population, followed by a survey covering a wide-range of health topics and behaviors including sexual behaviors, healthcare utilization, food insecurity, nutrition, reproductive health, substance use, MH and C-NCDs. The cohort also collects and stores participant’s blood samples to facilitate biomarker and genetic testing to answer current and future research questions. At enrollment, individuals are assigned a unique participant identification number. Biological specimens including blood for HIV, STIs and blood glucose level testing are collected and pre- and post-test counseling services are offered for all screening activities. The three APHS survey communities in Wakiso are all approximately 27 kilometers from Kampala, Uganda’s capital, and largest city. The present analysis utilizes cross-sectional data from round three of the APHS (conducted between 27^th^ Nov 2021– 23^rd^ Jan 2022). This sample was selected for several reasons. (1) Given the low prevalence of non-communicable diseases in Hoima district, we restricted our analytic sample to data from Wakiso district where the burden of C-NCDs was greater. (2) We wanted to characterize the burden of non-communicable diseases in the post-Covid-19 era. Data collection occurred after all pandemic related lockdowns had ended (which would limit the temporal generalizability) but should still capture the impact of the pandemic on non-communicable disease health outcomes. The present study received ethical approval and clearance from the Clark International University Research Ethics Committee (CIU-REC) and the Uganda National Council for Science and Technology (UNCST). Voluntary written informed consent was obtained before participants were enrolled into the study. Participants aged 13–17 years provided a written informed assent and their guardians provided a written informed consent for their participation. Additionally, voluntary written informed consent from witnesses of illiterate participants was also obtained for their participation.

### Measures and data collection

Following census activities, eligible participants are invited to enroll in APHS and provide informed consent before participating in the survey. Voluntary written informed consent was obtained from participants before they were enrolled into the study. Participants aged 13–17 years provided a written informed assent and their guardians provided a written informed consent for their participation. Additionally, voluntary written informed consent from witnesses of illiterate participants was obtained for their participation. Surveys are administered one-on-one, face-to-face by a same sex trained research staff member in the local language of Luganda.

Our primary exposures of interest were the presence of symptomology suggestive of depression or anxiety. The nine item Patient Health Questionnaire (PHQ-9) depression measure was used to screen for depressive symptomology [26] with a recall period of 2 weeks. Scores were summed across the nine scale items and categorical variable was created based on previously established and validated cut-offs [26]: no depressive symptomology (<5 points), mild depressive symptomology (5–9 points), moderate depressive symptomology (10–14 points) and severe depressive symptomology (> = 15 points). Presence of depressive symptoms (prevalence) was obtained by using a cut-off score of > = 5 points. For this analysis, the summed up continuous scores were used in the final analysis. We previously validated the PHQ-9 in this setting [27]. In the present study, the PHQ-9 had a Cronbach’s alpha of 0.81 which is consistent with this prior work and suggests strong internal consistency of the measure’s items in our sample.

The seven item Generalized Anxiety Disorder (GAD-7) measure was used to screen for anxiety symptomology [28] with a recall period of 2 weeks. Responses to scale items were measured on a scale of scores ranging from 0-Not all, 1- Several days, 2- More than half of the days to 3- Nearly every day. Scores were summed across responses and previously validated cut-off scores of 5, 10 and 15 were used to create the categories of mild, moderate—severe anxiety levels respectively [28]. Additionally, a dichotomous variable for any anxiety symptoms was created using a cut-off score of ≥5 in descriptive analyses. The summed up continuous scores for anxiety were used in the final analysis. The GAD-7 had strong internal consistency of 0.90. The GAD-7 was validated in an African context as fit for assessing anxiety symptomologies and produced a similar internal consistency of 0.86 by [29].

Our outcome of interest was the presence of at least one C-NCD. Participants were asked to respond in affirmative if they had ever been diagnosed with any of the following diseases: diabetes, hypertension, heart disease, cancer and stroke. A dichotomous variable was created where individuals replying “yes” to at least one C-NCD were categorized as a “yes” for C-NCDs.

Several self-reported sociodemographic characteristics selected a priori based on the existing non-communicable diseases literature were included in the present analysis such as age (continuous and categories into 35–44, 45–54, 55+ years), gender (male and female), education level (categorised into three: no schooling, primary school only, and secondary school through higher education), employment type (agriculturalists, housework, waitress/waiters, trading, and other (other employment types) and marital status (single and married). Community setting was categorized into three categories (semi-urban, rural and urban).

### Statistical analysis

Data analysis was performed using STATA software (Version 16.0, StataCorp LLC, College Station, TX, USA). Descriptive analyses were performed to characterize the study sample using frequencies for categorical variables and measures of central tendency for continuous variables. Bivariate analysis (with chi-square test or fisher’s exact as appropriate) was used to measure associations between sociodemographic variables (education level, marital status, employment type, age, gender and community setting), anxiety, and depression and C-NCD variables. Our outcome of interest, in the regression analysis was presence of any C-NCDs where our primary exposures of interest were depression and anxiety symptoms. Chi-square test was performed to test for differences in proportion between our dependent and independent variables. Those with p-value < = 0.25 were included in the final model. Measures of association (odds ratio), confidence intervals (CI) and p-values were interpreted in the unadjusted and adjusted logistic regression models.

## Results

### Sociodemographic characteristics of the study sample and presence of C-NCDs

The total number of participants in this study was 604. Table 1 presents the sociodemographic characteristics of study respondents stratified by presence or absence of at least one C-NCDs. Majority of respondents were females (63.6%, n = 384) with a median age of 46 years. The greatest proportion of participants were between 35–44 years of age (45%, n = 273), reported having ever been to school (and attained a primary level of education, 52.3%, n = 316), were from the semi-urban community (41.4%, n = 225), married (58.4%, n = 353) and worked in agricultural (36.1%, n = 218). In bivariate analysis (see Table 1), the prevalence of C-NCDs was significantly higher among females compared to males (24.2% vs 9%, P<0.001). Age was also significantly associated with presence of C-NCDs, with prevalence incrementally increasing in each of the age groups (34% vs 17.1% vs 11.4%, in the oldest, middle and youngest groups respectively, P<0.001). Marital status was also associated with presence of C-NCDs, participants who were not married at the time of this study had a significantly higher prevalence of C-NCDs compared to those that were married (24.3% vs 14.7%, P = 0.003). Overall, with the median age of 46 years, it indicated that individuals who were older than the median age are more likely to develop C-NCDs and the risk of developing C-NCDs increases as one gets older (see Table 1). Differences in C-NCDs were observed across different employment types and the prevalence of C-NCDs were higher among participants involved in housework (23.4%), 21.4% among waiters/ waitresses and 19% among agriculturalists and traders.

**Table 1:** Sociodemographic characteristics of the study sample and prevalence of C-NCDs among older adults.

Variable	N = 604 (Row %)				
		Presence of at least one C-NCD		*X* ^*2*^	P-Value
^ ^	n(%)	Yes 113 (18.7%)	No 491 (81.3%)		
**Gender**				21.0	<0.001
Male	220 (36.4)	20 (9)	200 (40.7)		
Female	384 (63.6)	93 (24.2)	291 (75.8)		
**Age median, [IQR]**	46 [39–54]	53[43–64]	45[38–53]	33.1	<0.001
35–44 years	273 (45.)	31 (11.4)	242 (88.6)		
45–54 years	181 (30)	31 (17.1)	150 (82.9)		
55 years +	150 (25)	51 (34)	99 (66)		
**Education level**				0.68	0.710
No Schooling	38 (6.3)	8 (21)	30 (78.9)		
Primary	316 (52.3)	62 (19.6)	254 (80.4)		
Secondary and above	250 (41.4)	43 (17.2)	207 (82.8)		
**Community Setting**				1.1	0.572
Semi Urban	225 (41.4)	46 (20.4)	179 (79.6)		
Rural	206 (34.1)	34 (16.5)	172 (83.5)		
Urban	173 (28.6)	33 (19.1)	140 (80.9)		
**Marital Status**				8.8	0.003
Single	251 (41.6)	61 (24.3)	190 (75.7)		
Married	353 (58.4)	52 (14.7)	30 (85.3)		
**Employment Type**				6.6	0.161
Agriculturalists	218 (36.1)	42 (19.3)	176 (80.7)		
Housework	124 (20.5)	29 (23.4)	95 (76.6)		
Waiters/waitress	42 (7)	9 (21.4)	33 (78.6)		
Trading	116 (19.2)	22 (19)	94 (81)		
Other*	104 (17)	11 (10.6)	93 (89)		

*Includes government worker, oil mining, fishing, students, medical worker and construction

### Distribution of C-NCDs by gender of the study sample

Table 2 presents the prevalence of individual C-NCDs, overall and stratified by gender. The disease with highest prevalence was hypertension (15.7%, n = 95) followed by diabetes with 4% (n = 24), and then stroke disease (1.7%, n = 10). The prevalence was significantly higher among females compared males (20.3% vs 7.7%, P<0.001), and was significantly higher among females compared to males (5.2% vs 1.8%, P = 0.04), with whole of the burden significantly occurring among females (2.6%, P = 0.016). Overall, the prevalence of C-NCDs was significantly higher among women (29.7% vs 10.5%, p<0.001). This held true when looking at each C-NCDs separately as well.

**Table 2:** Distribution of C-NCDs, stratified by gender (n = 604).

C-NCDs		Overall	Gender Stratified, n (Col %)		*X*^*2*^ or Fisher’s Exact	P-Value
		n(%)	Male	Female		
Diabetes					4.2	0.004
	Yes	24(4.0)	4(1.8)	20(5.2)		
	No	580(96.0)	216(98.2)	364(94.8)		
Hypertension					16.7	<0.001
	Yes	95(15.7)	17(7.7)	79(20.3)		
	No	509(84.3)	203(92.3)	306(79.7)		
Stroke					5.8	0.016
	Yes	10(1.7)	-	10(2.6)		
	No	594(98.3)	220(100)	374(97.4)		
Other NCDs					0.66	0.443
	Yes No	5 (0.8) 599 (99.2)	1(0.5) 219 (99.5)	4(0.1) 380(99.9)		
Overall C-NCDs Prevalence					21.0	<0.001
	Yes	113(18.7)	20(9.0)	93(24.0)		
	No	491(81.3)	200(91.0)	291(76.0)		

### Depression and anxiety

Table 3 describes the prevalence of depressive and anxiety symptomology in our sample as well as the presence of MH disorder and C-NCD comorbidities. Overall, 18.9% (n = 114) of participants had PHQ-9 scores indicative of depressive symptomology, with a median score = 2 and inter-quartile range (IQR) = 4 (upper quartile (Q3) = 4 and lower quartile (Q1) = 0). Three percent (3.2%) had symptoms indicative of moderate to severe depression. The prevalence of anxiety symptomology was 14% (n = 86), with a median GAD-7 score of 1 and IQR of 3, (upper quartile (Q3) = 3 and lower quartile (Q1) = 0). Nearly one-third of participants reported co-morbid depressive symptoms and C-NCDs (29%) or anxiety symptoms and C-NCDs (33%) while more than half of participants had co-occurring depressive and anxiety symptoms (71%).

**Table 3:** Overall prevalence of depressive and anxiety symptomology (n = 604).

	Freq, (%)
Prevalence of depressive symptomology *Median PHQ-9 score = 2 (IQR = 4)*	
No/Minimal depression	490 (81.1)
Mild depression	95 (15.7)
Moderate to Severe depression **Prevalence of depressive symptoms (Mild—Severe)**	19 (3.2) **114 (18.9)**
Prevalence of anxiety symptomology *Median GAD-7 score = 1 (IQR = 3)*	
No anxiety Mild anxiety Moderate to Severe anxiety	518 (86) 68 (11.3) 18 (3)
**Prevalence of anxiety symptoms (Mild—Severe)**	**86 (14)**
Prevalence of Comorbidities	
Depressive symptoms and C-NCD comorbidity	33 (29)
Anxiety symptoms and C-NCD comorbidity	28 (33)
Depression and Anxiety comorbidity	61 (71)

### Sociodemographic characteristics and experience of depression and anxiety symptoms

Table 4 explores unadjusted associations between experiences of anxiety and depression and various sociodemographic characteristics. Depressive symptomology was significantly associated with several key sociodemographic characteristics. Depressive and anxiety symptoms were more prevalent among women than men (25% vs 8.2%, 19% vs 5.5%, P<0.001) respectively. Level of education was also significantly associated with experience of depressive symptoms, with lower PHQ-9 scores among those with higher levels of education. Individuals residing in the semi-urban community had significantly higher prevalence of depressive (23.1%, P<0.005) and anxiety symptoms (19.6%, P = 0.006) compared to those from other community setting types. Professions reporting the highest prevalence of depressive symptoms were agriculture and housework (22.5% vs 23.4%, respectively, P<0.05), while individuals involved in trading and those working as waiters/waitresses reported the highest prevalence of anxiety symptoms (23%, 14.3%, P = 0.033) compared to individuals involved in other types of work.

**Table 4:** Depression and anxiety symptomology among adults aged 35 years and older by sociodemographic characteristics in Wakiso district in Uganda (n = 604).

	Experience of Depressive Symptoms (Row %)			Experience of Anxiety Symptoms (Row %)		
Variable	No depression: n = 490 (81.1%)	Presence of depression n = 114 (18.9%)	P-Value	No anxiety n = 518 (86%)	Presence of anxiety n = 86 (14%)	P-Value
**Gender**			<0.001			<0.001
Male	202 (91.8)	18 (8.2)		208 (94.6)	12 (5.5)	
Female	288 (75)	96 (25)		310 (81)	74 (19)	
**Age group**			0.149			0.932
35–44 years	230 (84.3)	43 (15.8)		233 (85)	40 (14.7)	
45–54 years	145 (80.1)	36 (19.9)		155 (86)	26 (14.4)	
55 years+	115 (76.7)	35 (23.3)		130 (87)	20 (13.3)	
**Education Level**			0.017			0.645
No Schooling	28 (73.7)	10 (26.3)		33 (87)	5 (13.2)	
Primary	246 (77.9)	70 (22.2)		267 (84.5)	49 (15.5)	
Secondary and above	216 (86.4)	34 (13.6)		218 (87)	32 (13)	
**Marital Status**			0.235			0.861
Married	292 (82.7)	61 (17.3)		302 (86)	51 (14.5)	
Single	198 (78.9)	53 (21.1)		216 (86)	35 (14)	
**Community Type**			0.046			0.006
Semi Urban	173 (76.9)	52 (23.1)		181 (80)	44 (19.6)	
Rural	167 (81.1)	39 (18.9)		188 (91)	18 (8.7)	
Urban	150 (86.7)	23 (13.3)		149 (86)	24 (14)	
**Employment Type**			0.057			0.033
Agriculturalists	169 (77.5)	49 (22.5)		190 (87)	28 (13)	
Housework	95 (76.6)	29 (23.4)		112 (90)	12 (9.7)	
Waitress/ Waiter	35 (83.3)	7 (16.7)		36 (85.7)	6 (14.3)	
Trading	98 (84.5)	18 (15.5)		89 (76.7)	27 (23)	
Others*	93 (89.0)	11 (10.6)		91 (87.5)	13 (12.5)	
**α = 0.05 level of significance**						

*Includes government worker, oil mining, fishing, students, medical worker and construction

### Association of C-NCDs with depressive and anxiety symptomology among adult individuals

In our multivariable models, we observed greater odds of experiencing C-NCDs among our older participants. Those 55 years and above had nearly five times greater odds of having a C-NCD compared to younger individuals (35 to 44 years of age) (AOR = 4.6, CI = 2.60–8.02) while individuals in the middle age group (45–54 years) had nearly two times greater odds of having a C-NCD compared to the younger adults (AOR = 1.70, CI = 0.97–3.05). In our adjusted models, individuals who were experiencing depressive symptoms had 12% greater odds of having a C-NCD compared to those without depressive symptoms (AOR = 1.12, CI = 1.10–1.20) (see Table 5). Being female was also associated with greater odds of having a C-NCD compared to males (AOR = 2.60, CI = 1.49–4.67). Table 6 presents unadjusted and multivariable results for the association between C-NCDs and experience of anxiety symptoms. Individuals who experienced any anxiety symptoms had higher odds of experiencing C-NCDs (AOR = 2.10, 95% CI: 1.21–3.70). As seen in the depressive symptom model, other factors significantly associated with increased odds of suffering from C-NCDs included participant age and gender with similar effect sizes observed.

**Table 5:** Logistic regression analysis for the association between C-NCD, depression and other sociodemographic characteristics.

Factor	Presence of at least one C-NCD	
	Unadjusted OR (95% CI)	Adjusted AOR (95% CI)
**Depression symptomology**		
Depressive symptoms	1.15 (1.12–1.23)	1.12 (1.10–1.20)***
**Gender**		
Male	1.0 (ref)	1.0 (ref)
Female	3.2 (1.91–5.35)	2.60 (1.49–4.67)***
**Age group (in years)**		
35–44	1.0 (ref)	1.0 (ref)
45–54	1.6 (0.94–2.76)	1.70 (0.97–3.05)
55+ years	4.02 (2.43–6.66)	4.6 (2.60–8.02)***
**Marital Status**		
Married	1.0 (ref)	1.0 (ref)
Single	1.85 (1.23–2.8)	1.12 (0.70–1.8)
**Community setting**		
Semi-urban	1.0 (ref)	1.0 (ref)
Rural	0.77 (0.47–1.26)	0.71 (0.42–1.20)
Urban	0.92 (0.56–1.51)	1.13 (0.62–1.88)
**Education level**		
No Schooling	1.0 (ref)	1.0 (ref)
Primary	0.92 (0.40–2.09)	0.97 (0.39–2.36)
Secondary and above	0.78 (0.33–1.82)	1.20 (0.49–3.07)

**Table 6:** Logistic regression analysis for the association between C-NCDs, anxiety and other sociodemographic characteristics.

Factor	Presence of C-NCDs	
	Unadjusted OR (95% CI)	Adjusted AOR (95% CI)
**Anxiety symptomology**		
Anxiety symptoms (suggestive of anxiety disorder)	2.46 (1.48–4.08)	2.1 (1.21–3.70)***
**Gender**		
Male	1.0 (ref)	1.0 (ref)
Female	3.20 (1.91–5.35)	2.91 (1.65–5.11)***
**Age group (in years)**		
35–44	1.0 (ref)	1.0 (ref)
45–54	1.61 (0.94–2.70)	1.74 (0.98–3.06)
55 years+	4.02 (2.43–6.65)	4.5 (2.61–7.88)***
**Marital Status**		
Married	1.0 (ref)	1.0 (ref)
Single	1.86 (1.23–2.80)	1.20 (0.73–1.86)
**Community setting**		
Semi Urban	1.0 (ref)	1.0 (ref)
Rural	0.77 (0.47–1.26)	0.73 (0.43–1.24)
Urban	0.92 (0.56–1.51)	1.00 (0.59–1.76)
**Education level**		
No Schooling	1.0 (ref)	1.0 (ref)
Primary	0.92 (0.40–2.09)	0.98 (0.40–2.38)
Secondary and above	0.78 (0.33–1.82)	1.13 (0.02–1.14)

*p<0.05

** p<0.01

*** p<0.001

## Discussion

The aim of this study was to characterize the burden of non-communicable diseases among older adults in Central Uganda and explore associations between MH disorders (anxiety, depression) and C-NCDs in this context. Overall, nearly one-fifth (18.7%) of study participants reported at least one C-NCD, which is substantially higher than previous estimates in Uganda (2.4%) [30]. The prevalence of any depression symptomology in this study was 18.9%. This finding was lower than estimates from Wakiso district (29.2%) obtained from work by [27] in Uganda but higher than recent estimates from the United States (where prevalence ranged from 7.5% to 14.2%) [31, 32]. Our observed prevalence is also lower than a recent meta-analysis of depression in Uganda which found a prevalence of 30%; however, a caveat for this comparison is that the meta-analysis included several samples comprised entirely of persons living with HIV, and these samples had the highest levels of depression, increasing the pooled prevalence estimate. More than one in ten (14%) participants had GAD-7 scores suggestive of mild to severe anxiety symptomology, which is comparable to prevalence estimates among adults (12.2%) in the United States [33] but much higher than prior estimates in the African region (5.3%) [34]. We observed gender differences in experiences of both depression and anxiety, with our results suggesting that women are disproportionately burdened by both issues and should be a priority population for mental health screening and services.

Some of our findings aligned with estimates from the global literature, while others differed. The prevalence of depression, for instance, was much higher in our study (18.9%), compared to nationally representative samples from the United States (14.2%) [31, 32]. The anxiety findings from our study, were consistent with findings from other settings. However we observed a substantially lower prevalence of both depression and anxiety symptoms than estimates from Africa during the Covid-19 pandemic, which suggests that conditions may have improved by the time of our data collection [35]. Similarly, our findings on gender disparities in prevalence of MH disorders are consistent with global trends [36]. Extant literature offers several explanations for this. Women may experience higher rates of depression and anxiety (attributable to biological and social factors) [37], they may be more likely to self-report MH challenges leading to response bias, and they may be more likely to recognize mental distress due to cultural and gender norms or a combination of these factors [38]. Data from the global burden of disease literature suggests that gender disparities in depression increase over the lifespan, suggesting that early intervention among women may reduce this disparity among older adults [36]. C-NCDs were also more prevalent among women than men in our sample. Our observed gender differences in the prevalence of C-NCDs is also consistent with global trends [39] and is likely attributable to a greater number of risk factors including higher rates of obesity and MH disorders [40].

We observed significant associations between both depression and anxiety and experience of C-NCDs suggesting high multi-morbidity of NCDs in this setting. While we are unable to disentangle the temporal relationship between C-NCDs and MH in this cross-sectional analysis, extant literature suggests a bidirectional relationship between physical and mental well-being with prior studies demonstrating a temporal relationship in both directions. Some studies have found that C-NCDs cause depression and anxiety [41]. For example, depression and cardiovascular risk are strongly correlated, many patients with C-NCDs have MH disorders [42]. Other studies have shown that depression and anxiety symptoms increase the risk of C-NCDs or exacerbate the condition of patients with C-NCDs. Depression and anxiety disproportionately burden those with chronic medical illnesses and worsen the outcomes of many medical illnesses [24]. The high prevalence of C-NCDs in this sample suggests that screening for all three issues should be integrated into routine healthcare, both to address and intervene on them as individual and interrelated health issues. Findings around age and sex indicate that older persons and women in particular would benefit from universal screening.

The study had several strengths and limitations. The cross-sectional nature of the study precludes our ability to infer causality. We also report MH screening results and not diagnostics. A further limitation of our measures was our reliance on self-report for the presence of C-NCDs which increases risk of bias. Finally, since the study was conducted in a single district (Wakiso), we acknowledge that this may limit generalizability. However, we utilize data from a large population-based cohort design which includes communities specifically selected because they capture the heterogeneity of Uganda’s community types, strengthening our ability to infer trends in the broader Ugandan context.

## Conclusions

Accurate estimates generated from population-based studies on the burden of C-NCDs and MH symptomology are critical in mobilizing and allocating resources and providing evidence for scaling-up and integration of C-NCDs and MH interventions in the country. In SSA and Uganda particularly, research in C-NCDs and MH is limited [43] which compromises design of advanced interventions targeting affected populations. Additionally, the C-NCDs and MH services are still very limited and the small public health budget for C-NCDS and MH services often compromises scale-up and quality of service provision. Understanding and quantifying the burden of C-NCDs and MH can help inform and facilitate re-allocation of resources/funds to meet the public health need. Findings from the present study adds to the existing state of knowledge on C-NCDs and its association with MH symptoms which importantly informs the need to integrate of MH, C-NCDs screening and diagnosis services and design of targeted interventions to the vulnerable populations in the country. From the policy perspective, the evidence reviewed here supports the inclusion of MH as one of the “big five” diseases in the non-communicable disease prevention strategies as well as the sociodemographic characteristics as key risk factors. This is critical for re-directing and designing appropriate interventions for most at risk and vulnerable populations in the country.
